# Magnetic enrichment–coupled flow cytometry for highly sensitive detection of persistent CAR-T cells in spiked and clinical peripheral blood samples

**DOI:** 10.3389/fimmu.2026.1872290

**Published:** 2026-10-21

**Authors:** Loubna Abdel Hadi, Gokul Thangamani, Samira Sheikh, Syed Mazher Hussain, Alvin Jon Ingel, Shahid Imtiaz Ahmed, Antonio Alfonso Bencomo Hernandez, Fatima Mohammed Al Kaabi, Yendry Ventura Carmenate, Rupert Handgretinger

**Affiliations:** 1 Research and Development Department, Abu Dhabi Stem Cells Center, Abu Dhabi, United Arab Emirates; 2 Yas Clinic Khalifa City (YCKC) Hospital, Abu Dhabi, United Arab Emirates; 3 Department of Pediatrics, Yong Loo Lin school of Medicine, National University of Singapore, Singapore, Singapore; 4 Director Office, Emirates Drug Establishmnet (EDE), Masdar city, Abu Dhabi, United Arab Emirates

**Keywords:** CAR-T, magnetic enrichment and flow cytometry, persistence, phenotyping, single cell analysis

## Abstract

**Background:**

Long-term monitoring of chimeric antigen receptor T-cell (CAR-T) persistence is essential for assessing therapeutic efficacy, particularly when circulating CAR-T cell levels decline following infusion. Although quantitative real-time PCR (qPCR) provides highly sensitive detection of CAR transgenes, it lacks single-cell resolution, phenotypic characterization, and the ability to assess cell viability.

**Methods:**

In this study, we adapted and validated an automated enrichment-mode on MACSQuant flow cytometry that integrates automated immunomagnetic enrichment with multiparametric flow cytometric analysis to enhance the sensitivity of detecting and quantifying rare CAR-T cell populations. The assay’s limit of detection and potential clinical utility were evaluated using CAR-T cell spike-in experiments in peripheral blood mononuclear cell (PBMC) backgrounds and clinical blood samples obtained from patients who received CD19-targeted CAR-T cell therapy. Results were compared with a complementary qPCR assay for vector copy number (VCN) detection.

**Results:**

The enrichment-based workflow enabled same-day detection of viable CAR-T cells by selectively enriching CAR-T cells while excluding non-target peripheral blood leukocytes, thereby substantially improving detection sensitivity at very low cell frequencies in post-infusion patient samples. Concordant CAR-T cell detection was observed between the enrichment-coupled flow cytometry workflow and qPCR. While qPCR served as an orthogonal approach for sensitive transgene detection, the enrichment-based flow cytometry workflow provided additional single-cell information, including viable CAR-T cell frequency and phenotypic characterization.

**Conclusion:**

By combining enhanced sensitivity with single-cell and functional resolution, this enrichment-coupled flow cytometry workflow complements qPCR-based genomic detection and may serve as a rapid, accurate, and clinically valuable tool for the longitudinal monitoring of CAR-T cell persistence in patients receiving CAR-T cell therapy.

## 1 Introduction

Chimeric antigen receptor T-cell (CAR-T) therapy has transformed the management of relapsed or refractory B-cell malignancies, producing durable remissions in patients who previously had limited options (1, 2). Long-term disease control after CAR-T infusion closely depends on the persistence and functional competence of the infused cells. Therefore, several studies and recent consensus guidelines emphasize sustained monitoring of *in vivo* CAR-T persistence over the medium and long term therapeutic effects because prolonged detectability correlates with improved event-free and overall survival of patients (3, 4). B cell aplasia (BCA) remains a clinically useful surrogate for ongoing anti-CD19 CAR-T activity (5), but direct quantification of circulating CAR-T cells provides a more detailed, cell-level view of *in vivo* kinetics and immune reconstitution in patients that can lead to better clinical decision-making.

The peripheral blood kinetics of infused CAR-T cells typically follow a biphasic pattern: an early antigen-driven expansion of these cells peaking within the first one to two weeks, a subsequent contraction, and finally a prolonged low-level persistence driven in part by differentiation into memory-like T-cell phenotypes (6). The magnitude and durability of these phases are functionally meaningful; early expansion is linked to initial cytokine- release syndrome (inflammatory cytokines) and tumor control, while prolonged low-level persistence is associated with ongoing tumor surveillance and long-term remission (7, 8). Despite the clinical importance of persistence, standardized diagnostic tools and consensus guidelines for post-infusion CAR-T monitoring remain limited.

Molecular approaches such as quantitative PCR (qPCR) and next-generation sequencing (NGS) offer high analytical sensitivity for detecting CAR transgene sequences and minimal residual disease, but they assess bulk nucleic acid and cannot directly report single-cell viability and immunophenotypes of circulating CAR-T cell post-infusion (9). Multiparametric flow cytometry provides data at single-cell resolution and allows simultaneous phenotypic characterization of viable CAR-T cells, enabling rapid, same-day assessment of lineage, differentiation state, and status of immune reconstitution. However, conventional flow cytometry can lack the sensitivity needed to detect ultra-rare CAR-T populations in complex clinical samples because of background noise and event discrimination challenges (10).

Immunomagnetic pre-enrichment of CAR-T cells from patient blood samples can overcome these limitations by selectively concentrating target cells before flow-cytometric acquisition. This approach enhances detection sensitivity while preserving phenotypic characterization of the enriched CAR-T cell population and minimizing the loss of rare target cells that may otherwise remain undetectable during conventional flow-cytometric analysis (11, 12). Coupling automated magnetic enrichment with high-parameter flow cytometry on a fully integrated platform (e.g., MACSQuant^®^ Enrich-sort-analyse workflow, Miltenyi Biotech) provides a scalable, standardized solution for CAR-T monitoring with minimal hands-on manipulation during enrichment, thereby reducing operator-dependent variability and improving workflow reproducibility. In this study, we leveraged the MACSQuant automated magnetic-enrichment platform to develop a streamlined assay capable of detecting rare CAR-T cells in peripheral-blood mononuclear cell samples (PBMCs). Specifically, we aimed to *(i)* validate the analytical sensitivity and reproducibility of the enrichment-plus-flow-cytometry workflow using serial spike-in dilutions of CAR-T cells in PBMCs, *(ii)* evaluate its analytical performance using qPCR as an additional method for confirmation of CAR transgene detection, and *(iii)* apply the methodology to clinical samples to address the feasibility of establishing a practical tool for longitudinal CAR-T monitoring.

## 2 Materials and methods

### 2.1 Isolation of peripheral blood mononuclear cells

PBMCs were obtained from de-identified healthy donors by apheresis using the Spectra Optia^®^ system (Terumo BCT, Inc., software version 11.0) with the MNC Collection program. Tumor CD19- or BCMA-antigen targeting CAR-T cells were generated from the same donor pool using the CliniMACS Prodigy^®^ platform with the T Cell Transduction (TCT) software and lentiviral vectors (Miltenyi Biotec). For spike-in experiments, the genetically engineered CAR-T cells products were spiked into 10 × 10^6^ PBMCs at 10^6^, 10^5^, 10^4^, 10³, and 10² cells, corresponding to final frequencies of CAR-T cell products as 10%, 1%, 0.1%, 0.01%, and 0.001%, respectively. A CAR-negative PBMC sample containing no spiked CAR-T cells was included as a blank control to assess background signal and assay specificity. Following preparation of each dilution and Blank control, samples were divided into two aliquots: one aliquot was analyzed by flow cytometry with and without immunomagnetic enrichment, while the second aliquot was used for genomic DNA extraction and vector copy number (VCN) quantification by qPCR. This parallel analysis was performed to provide additional confirmation of CAR-T cell detection across the tested dilution range and to evaluate assay performance relative to a CAR-negative background.

For clinical sample analysis, peripheral blood samples were obtained from 10 patients who had previously received CD19-directed CAR-T cell therapy as part of routine post-infusion follow-up monitoring. Samples were selected to represent different stages of CAR-T cell persistence and were grouped according to the observed CAR-T cell frequency. Samples with CAR-T cell frequencies greater than 10% were collected approximately 15 days’ post-infusion, samples with frequencies between 1% and 10% were collected approximately 90 days’ post-infusion, samples with frequencies between 0.1% and 1% were collected approximately 180 days’ post-infusion, and samples with frequencies between 0.01% and 0.1% were collected approximately 360 days’ post-infusion. Three EDTA-anticoagulant tubes containing of peripheral blood each were pooled and processed within 24 hours of collection. Prior to PBMCs isolation, a complete blood count (CBC) was performed to determine leukocyte and lymphocyte counts. 5 mL Blood samples were diluted 1:2 with phosphate-buffered saline (PBS) and layered onto equal number of Ficoll-Hypaque density gradient medium (Histopaque -1077, ρ = 1.077 g/mL, Sigma, Cat. # 10771). Following centrifugation at 800 × g for 30 minutes at room temperature without brake, PBMCs layer at the plasma–Ficoll interface was carefully collected. The recovered PBMCs were washed with PBS and centrifuged at 1,000 × g for 10 minutes at room temperature. Cell pellet was suspended in 100 µL and subsequently stained and processed according to the CAR-T cell detection and enrichment protocol described below.

### 2.2 Detection of chimeric antigen receptor

For CAR detection, 10 μL of biotinylated CD19 or BCMA CAR Detection Reagent (Miltenyi Biotec, Cat. # 130-115-965) was added to 100 µL of PEB buffer (PBS, pH 7.2, 2 mM EDTA, 0.5% HSA) containing the cell mixture with or without CAR-T and incubated for 10 minutes at room temperature. After two washes with PEB buffer, cells were stained with anti-biotin-PE (2 μL, Cat. # 130-110-951), anti-CD3 (2 μL, Cat. # 130-113-138), anti-CD45 (2 μL, Cat. # 130-090-756), and 7-AAD (10 μL, Cat. # 130-111-568) in 100 µL PEB buffer for 10 minutes at room temperature in the dark or with the isotype REA Control Antibody, human IgG1-PE (2 μL, Cat. # 130-1130-450) for assessing non-specific binding. Cells were washed and incubated with anti-PE microbeads (10 μL, Cat. # 130-048-801) in 90 µL PEB buffer for 10 minutes at 4 °C. All antibodies used in this assay were from Miltenyi Biotech. After a final wash, cells were resuspended in 500 µL PEB buffer for acquisition as follows.

### 2.3 CAR-T cell sample acquisition and data analysis

CAR-T cell analysis was performed using the automated enrichment, EnrichS acquisition mode on the MACSQuant Analyzer 10 (Miltenyi Biotec, Cat. # 130-096-343) equipped with an integrated MACSQuant Enrichment Column (Cat. # 130-094-458). This mode incorporates immunomagnetic separation directly into the instrument workflow, enabling automated pre-enrichment of low-frequency target cells prior to flow cytometric acquisition. For each sample, 100 µL was acquired directly using the standard flow cytometry mode unenriched (UnEnr), whereas 400 µL was processed using the EnrichS workflow prior to acquisition. During enrichment, magnetically labeled CAR-T cells are retained within the separation column while the majority of unlabeled mononuclear cells are depleted, resulting in a substantial increase in the frequency of target cells available for analysis. A detailed description of the workflow steps, processing times, and sample uptake volumes for both the UnEnr and EnrichS modes is provided in **Supplementary Table S1**. Enriched cells were analyzed within the same acquisition sequence using the CAR-T Cell Express Mode, specifically the CAR-T cell transduction h-02 software, which applies predefined gating strategies and standardized analysis templates for reproducible identification and characterization of populations of CAR-T cells. Multicolor compensation was first established by using single-stained controls, and was automatically calculated by the instrument software. Immune Cell Composition compensation and CAR-T Transduction compensation using the StainExpress Mode, have been performed ensuring accurate resolution of overlapping fluorochromes and consistent identification of CAR-expressing T cells within complex immune subsets. CAR-T cell frequency was calculated as the percentage of viable CD45^+^CD3^+^ parent cells, and the gating strategy is presented in **Figure 1**.

**Figure 1:**
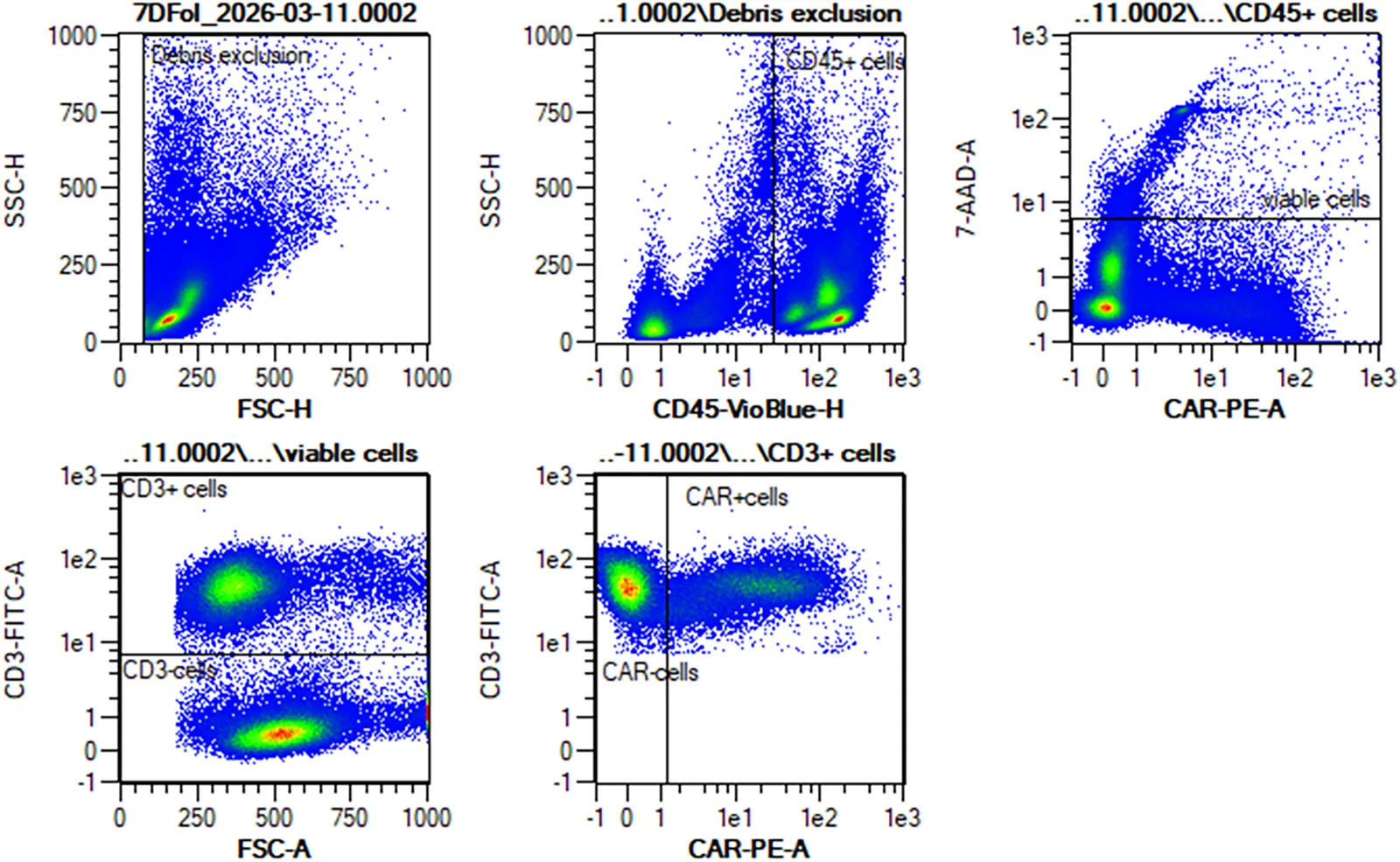
Gating strategy for CAR T-cell detection in UnEnr and EnrichS modes. Sequential gating was employed to identify target populations within both standard and enriched acquisition modes using mainly the CAR-T-Cell-Transduction-h-02 panel. Briefly, following the exclusion of debris and cell doublets using forward and side scatter characteristics, initial events were defined by CD45^+^ expression. Viable T cells were subsequently identified by 7AAD negative expression. Within the CD3+ T-cell gate, the CAR-positive populations (CD19- or BCMA-directed) were identified based on the expression of Biotin-PE, utilizing a specific biotin-conjugated CAR detection reagent. This hierarchical gating strategy was applied consistently to both UnEnr - samples and those processed via the EnrichS mode to ensure comparative accuracy.

### 2.4 Absolute quantification of CAR-T cells (cells/mL) in patient samples

For the determination of Absolute CAR-T cell counts, the MACSQuant Analyzer 10 employs volumetric counting technology, which enables the direct measurement of absolute cell counts without the need for counting beads or external reference standards. First, the absolute number of viable CD3^+^ T cells was obtained by direct volumetric analysis of 100 µL of whole blood stained with the StainExpress™ Immune Cell Composition Cocktail, human (Miltenyi Biotec, Cat. No. 130-127-637), according to the manufacturer’s instructions. For patient samples, after verification of the CAR-specific signal in the corresponding auto-enriched fraction, the absolute CAR-T cell count was calculated from the non-enriched sample by multiplying the frequency of viable CAR-T cells within the viable CD3^+^ T-cell population by the absolute CD3^+^ T-cell count. To account for background staining, the percentage of non-specific events detected in the corresponding blank control was subtracted from the measured CAR-T cell frequency before calculation. In addition to reporting CAR-T cell frequencies as a percentage of viable CD3+ T cells, absolute CAR-T cell counts were determined to provide a more accurate assessment of CAR-T cell persistence, particularly in samples containing low numbers of circulating lymphocytes.

### 2.5 Determination of limit of detection and limit of quantification

Following enrichment and flow-cytometric acquisition, the limit of detection (LOD) and limit of quantification (LOQ) were determined based on the lowest concentration at which CAR-T cells could be reliably detected and quantified with acceptable precision. The coefficient of variation (CV) was calculated and the functional LOD was defined as the lowest spike-in concentration consistently distinguishable from the blank control, while the LOQ was defined as the lowest concentration that could be quantified with acceptable assay precision (CV ≤ 20%).

### 2.6 DNA extraction and quantitation

DNA was isolated by using QIAamp DNA Blood Kits for Genomic DNA Extraction (Cat. # 51106, Qiagen), and by following manufacturer’s guidelines. In the final step, the DNA was eluted out in 100 µL nuclease-free water, and quantitated in Nanodrop spectrophotometer (DeNovix DS-11).

### 2.7 Quantitative real-time PCR copy vector number determination

The qPCR assays for determination of VCN in DNA samples from PBMCs spiked with CAR-T cell products were carried out by using MACS^®^ COPYcheck Kit, human (Cat. # 130-128-157) and TaqMan Fast Advanced Master Mix for qPCR (Cat. # 4444557 Applied Biosystems/ThermoFisher Scientific) by following manufacturer’s instructions. Master reaction mix along with primers and probe mix for required number of qPCR assays was calculated and pipetted out, and an aliquot of 50 µL of nuclease-free water to serve as no template control (NTC) were separately prepared in PCR workstation. Briefly, 50 µL of each test sample (600 ng DNA), no template control (negative control), and standards (pLTG2713, also serve as positive controls) were separately mixed with 50 µL TaqMan Fast Advanced Master Mix with primers and probes, and quantitative PCR (qPCR) assays were carried out for each sample in triplicate in 25 µL reaction volumes in a 96-well plate. The plate was covered with a plastic sealing film, briefly centrifuged to collect the reaction volume at the bottom of the wells, and qPCR assays were carried out in LightCycler 480 II (Roche). The thermal cycling regimen was programmed for 50 °C for 120 sec. x 1 cycle, 95 °C for 20 sec. x 1 cycle, 95 °C for 5 sec./56 °C for 20 sec./65 °C for 20 sec. x 40 cycles followed by 65 °C for 20 sec., and plate reading for data collection.

### 2.8 Statistical analysis

Statistical analyses were performed using GraphPad Prism software (version 8.4.3; GraphPad Software, San Diego, CA, USA). Data normality was assessed using the Shapiro–Wilk test. As the analyzed datasets met the assumptions of normality, comparisons were performed using a two-way repeated-measures analysis of variance (ANOVA) to evaluate the effects of CAR-T cell frequency and enrichment on assay performance. Descriptive statistics are presented as mean ± standard deviation (SD), and exact p-values are reported where applicable. Statistical significance was defined as ****, p < 0.0001.

## 3 Results

### 3.1 Automated immunomagnetic enrichment significantly enhances the analytical sensitivity of the MACSQuant Analyzer for high-resolution detection of low-frequency CAR-T cells

To validate the sensitivity and reproducibility of the automated immunomagnetic enrichment platform, spike-in experiments were conducted by using human PBMCs from healthy donors. Across a wide range of target frequencies, the system demonstrated a marked and consistent enrichment of both CD19- and BCMA-CAR-T cells (**Table 1**). Prior to enrichment, the baseline frequencies in the UnEnr- samples closely matched the theoretical input ratios, spanning a four-log range from 10% (10^7^:10^6^) down to 0.001% (10^7:^10^2^). Following automated enrichment (AutoEnr), both, the CD19- as well as BCMA- CAR-T cell populations exhibited a substantial and statistically significant increase in detectable frequency across all dilutions. At the highest input ratios (10^7^:10^6^) and (10^7^:10^5^), enrichment yielded high-purity populations approaching ~90% for both constructs, indicating efficient enrichment followed by a significant enhancement of the signal. Notably, robust performance was maintained even at extreme dilutions; samples with baseline frequencies as low as 0.1–0.01% were enriched resulting in a marked increase of the CAR-T signal to approximately 20 to 7-9% respectively. Even at the rarest condition tested (0.001% input), the target cells were concentrated to a clearly quantifiable signal of ~1.4–1.7% within the analyzed fraction. Importantly, cell viability remained consistently high throughout the enrichment process, ranging from 95.34% to 98.76%, indicating that automated immunomagnetic enrichment did not adversely affect cell integrity (data not shown). In addition, only negligible CAR-positive background events were observed in the CAR-negative PBMC blank control samples (**Supplementary Figure S1**). The mean background signal across blank controls was 0.000075% and 0.000069%. for CD19 and BCMA-CAR, respectively. Similarly, isotype control staining produced only negligible background signals (~0.0001%), which remained below the assay detection threshold and were not detectable following enrichment (**Supplementary Table S2**; **Supplementary Figure S1**). Statistical analysis confirmed that the automated workflow significantly increased CAR-T cell frequencies across all experimental conditions (***P<0.0001). Furthermore, the enrichment performance remained consistent between the CD19- and BCMA- CAR-T cells, suggesting that the platform’s utility is independent of expression specific CAR. Representative flow cytometric dot plots in **Figure 2** illustrate the transition of CD3^+^/Biotin^+^ events from near-invisible populations in the UnEnr samples (left row) to well-defined, robust clusters following automated enrichment, AutoEnr (right row).

**Table 1:** Frequency of CD19- and BCMA- CAR T Cells before and after automated PE based magnetic enrichment of viable cells.

Experimental groups	PBMCs: CAR T-cell ratio				
	10^7^: 10^6^ (10%)	10^7^: 10^5^ (1%)	10^7^: 10^4^ (0.1%)	10^7^: 10^3^ (0.01%)	10^7^: 10^2^ (0.001%)
UnEnr					
CD19- CAR-T	10.28 ± 2.61	1.19 ± 0.39	0.16 ± 0.06	0.020 ± 0.004	0.0025 ± 0.00001
BCMA- CAR-T	9.67 ± 1.45	1.07 ± 0.24	0.12 ± 0.03	0.015 ± 0.003	0.0017 ± 0.00001
AutoEnr ***					
CD19- CAR-T	88.83 ± 6.38	46.32 ± 3.14	20.26 ± 1.07	6.42 ± 0.91	1.66 ± 0.05
BCMA- CAR-T	91.54 ± 4.27	51.22 ± 2.79	19.43 ± 2.32	8.67 ± 0.52	1.38 ± 0.03

PBMC samples spiked with CD19- or BCMA-CAR T cells at defined concentrations ranging from 0.001% to 1%. CAR^+^ T-cell frequencies were measured by flow cytometry in UnEnr- and AutoEnr-samples. Data are presented as mean % ± SEM of CD3+CD19 or BCMA-CAR-Biotin-PE from three independent experiments with three different blood donors. Statistical significance was defined as ****p < 0.0001.

**Figure 2:**
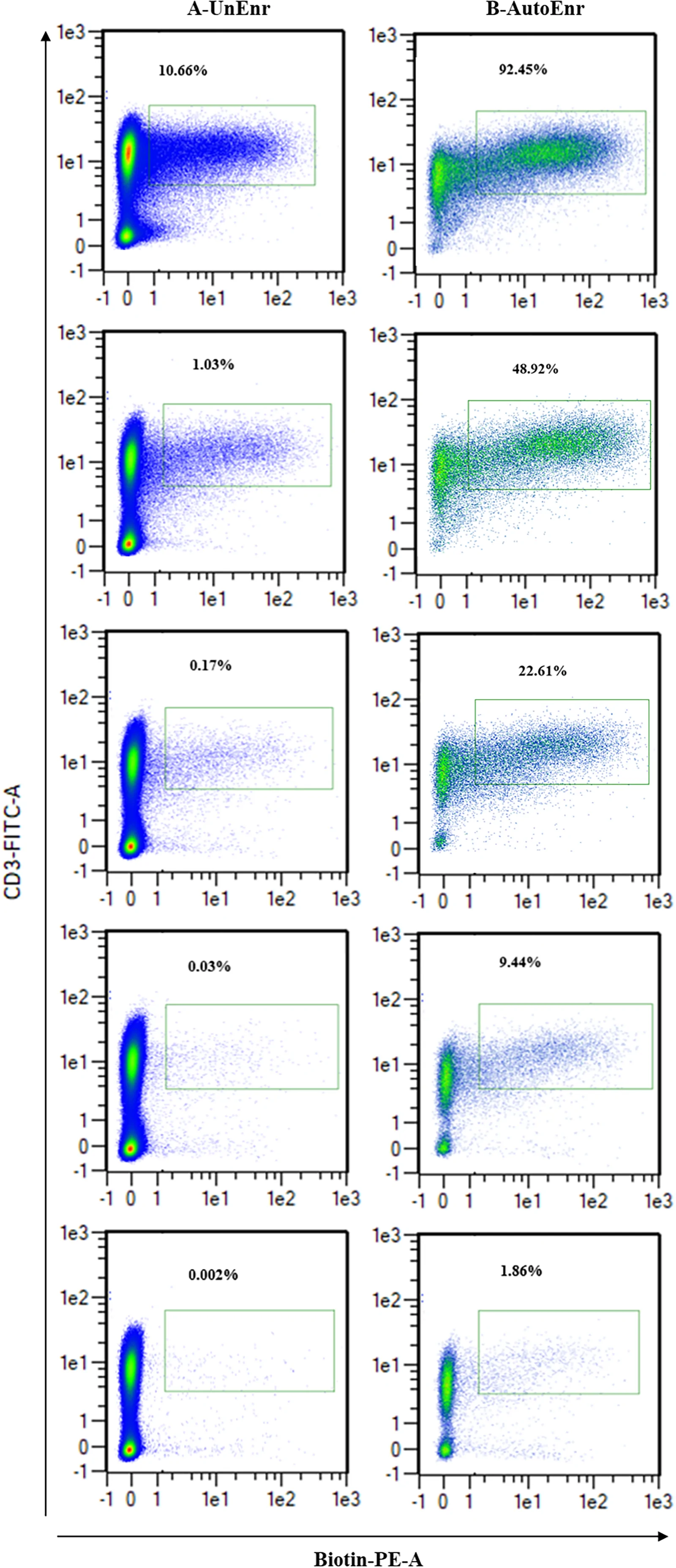
High-Resolution Detection of CD19-CAR-T Cells Pre- and Post-Automated Immunomagnetic Enrichment. Representative flow cytometric dot plots illustrate the identification of CD19-CAR-T cells within healthy donor PBMC samples across a series of defined spike-in concentrations (0.001% to 10%). Panel A shows samples UnEnr, while the panel B demonstrates the same populations following automated PE-based magnetic enrichment, AutoEnr. Frequency values shown within the plots represent the percentage of CD3/CAR-Biotin-PE events as a fraction of the total CD3- T-cell population. Data are representative of three independent experiments performed using three different blood donors.

### 3.2 qPCR assay for determination of vector copy number in DNA from PBMCs spiked with CAR-T cell products detects CAR-Tcells at low frequencies

qPCR assay for detection of VCN in DNA samples from PBMCs spiked with CAR-T cell products engineered with CD19- or BCMA- CAR expressing vector demonstrated the level of CAR-T cell detection of vector Gag DNA sequence at a CAR-T to PBMCs ratio of 1:100000 in two out of three spiking experiments (**Table 2**) supporting the sensitivity of detection of CAR-T cells. This lower limit of detection by using DNA samples from PBMCs spiked with CAR-T cells as determined by qPCR VCN assay matched to the Flow Cytometry data from unenriched PBMCs (10^7^) spiked with CAR-T cells (10^2^) at a ratio of 10^7^:10^2^ respectively showing the lower limit of detection of VCN as 0.0098 in CD19-CAR-T cells, and as 0.016 in BCMA-CAR-T cells. The “not detected” result observed in one of the three qPCR experiments at the 0.001% level likely reflects stochastic sampling effects at ultra-low target frequencies close to the assay’s limit of detection. At this dilution range, minor variations in cell distribution during aliquoting and DNA extraction, can lead to intermittent absence of detectable template despite equivalent theoretical input. This explains the variability observed between replicate spiking experiments and does not indicate a lack of assay sensitivity, but rather inherent sampling limitations at the lowest tested concentration.

**Table 2:** The detection of VCN at five log dilutions of CAR-Tcells with PBMCs in two out of three experiments presented is mentioned as limit of detection shows the limit of detection as 1 in 100000 cells.

CAR	Spiking CAR-T cells %	CAR-T cell:PBMCs ratio	VCN/CAR-T cell
CD19	10	1:10	0.64
CD19	1	1:100	0.12
CD19	0.1	1:1000	0.032
CD19	0.01	1:10000	0.012
CD19	0.001	1:100000	Not detected
CD19	10	1:10	2.78
CD19	1	1:100	0.52
CD19	0.1	1:1000	0.066
CD19	0.01	1:10000	0.024
CD19	0.001	1:100000	**0.0098**
BCMA	10	1:10	1.62
BCMA	1	1:100	0.4
BCMA	0.1	1:1000	0.04
BCMA	0.01	1:10000	0.025
BCMA	0.001	1:100000	**0.016**

### 3.3 Automated immunomagnetic enrichment enables high-sensitivity detection of rare CAR-T cells in post-infusion patient samples

To evaluate the clinical utility of the platform, peripheral blood mononuclear cell (PBMC) samples from 10 patients were analyzed during follow-up study after CD19-CAR-T cell infusion. Consistent with the results observed with PBMCs from healthy donors and CAR-T cells spike-in experiments, automated immunomagnetic enrichment of CAR-T cells from patients divided into four categories (≥10%, 1-10%, 0.1-1%, and 0.01-0.1%) provided a marked and reproducible increase in the detectable frequency of CAR-T cells across all baseline abundance ranges, including samples approaching the lower limit of conventional flow cytometric detection. Automated enrichment resulted in a statistically significant expansion of the CAR-T-cell fraction across all tested groups (**Table 3**). In samples with a baseline frequency of ≥10%, enrichment increased the mean CAR-T population from 12.9% ± 2.1% to 88.9% ± 5.7% (paired t test, P < 1 × 10^-^¹^0^). Similarly, significant increases were achieved in samples with intermediate baseline frequencies of 1–10% (2.0% ± 0.8% vs 53.9% ± 4.9%, P < 1 × 10^-^¹^0^) and 0.1–1% (0.44% ± 0.20% vs 23.0% ± 1.8%, P < 1 × 10^-^¹^0^). Notably, the platform demonstrated exceptional sensitivity in samples with extremely low baseline frequencies (0.01–0.1%), where the CAR^+^ fraction was enriched from 0.034% ± 0.013% to 5.82% ± 1.16% (P < 1 × 10^-^¹²). These findings confirm that the automated workflow enables the robust identification and quantification of circulating CAR-T cells in post-infusion patient monitoring, providing high-resolution data even when target cells are present at near-undetectable baseline levels. Representative flow cytometric dot plots and corresponding fluorescence-intensity histograms in **Figure 3** illustrate the high-resolution detection of these rare circulating CD19-CAR-T cells in the peripheral blood of post-infused patients. In addition, **Table 4** reports the absolute counts of circulating CAR-T cells calculated from non-manipulated whole blood samples, based on the total CD3^+^ T-cell concentration measured using the StainExpress™ Immune Cell Composition Cocktail. These calculations were performed only after confirmation of CAR-specific signal in the AutoEnr samples, and therefore reflect the true *in vivo* frequency of CAR-T cells within the native CD3^+^ compartment without any enrichment or sample manipulation.

**Table 3:** Peripheral blood mononuclear cell samples were obtained as follow-up samples from patients who had previously received CD19-directed CAR T-cell.

	CD19-CAR- T-cell frequency (%)							
	≥ 10%		1%-10%		0.1-1%		0.01-0.1%	
Follow-up samples	UnEnr (%)	AutoEnr (%) ****	UnEnr (%)	AutoEnr (%) ****	UnEnr (%)	AutoEnr (%) ****	UnEnr (%)	AutoEnr (%) ****
PT1	13.44	90.56	2.77	58.33	0.18	26.12	0.021	4.32
PT2	13.76	82.01	2.03	59.83	0.51	25.16	0.021	6.41
PT3	17.02	76.56	2.16	53.28	0.63	23.34	0.032	6.65
PT4	10.66	88.61	3.14	57.84	0.52	21.76	0.051	5.36
PT5	10.47	91.23	1.54	54.23	0.19	25.62	0.031	4.31
PT6	11.34	94.26	1.42	57.89	0.23	21.88	0.025	7.34
PT7	14.56	87.43	3.18	47.86	0.42	22.73	0.034	5.56
PT8	12.87	91.84	1.08	49.63	0.75	22.45	0.057	4.56
PT9	10.03	90.37	1.16	54.27	0.36	21.06	0.023	6.65
PT10	10.54	89.91	1.43	46.73	0.58	24.03	0.043	7.12
**Mean ± SD**	**12.9 1± 2.11**	**88.92 ± 5.70**	**2.00 ± 0.80**	**53.91 ± 4.91**	**0.44 ± 0.20**	**23.01 ± 1.80**	**0.034 ± 0.013**	**5.82 ± 1.16**

CAR^+^ T-cell frequencies were determined by flow cytometry in UnEnr and AutoEnr-samples. Data represent individual patient samples (PT1–PT10) and are summarized as mean ± SD in bold. Statistical significance was defined as ****p < 0.0001.

**Figure 3:**
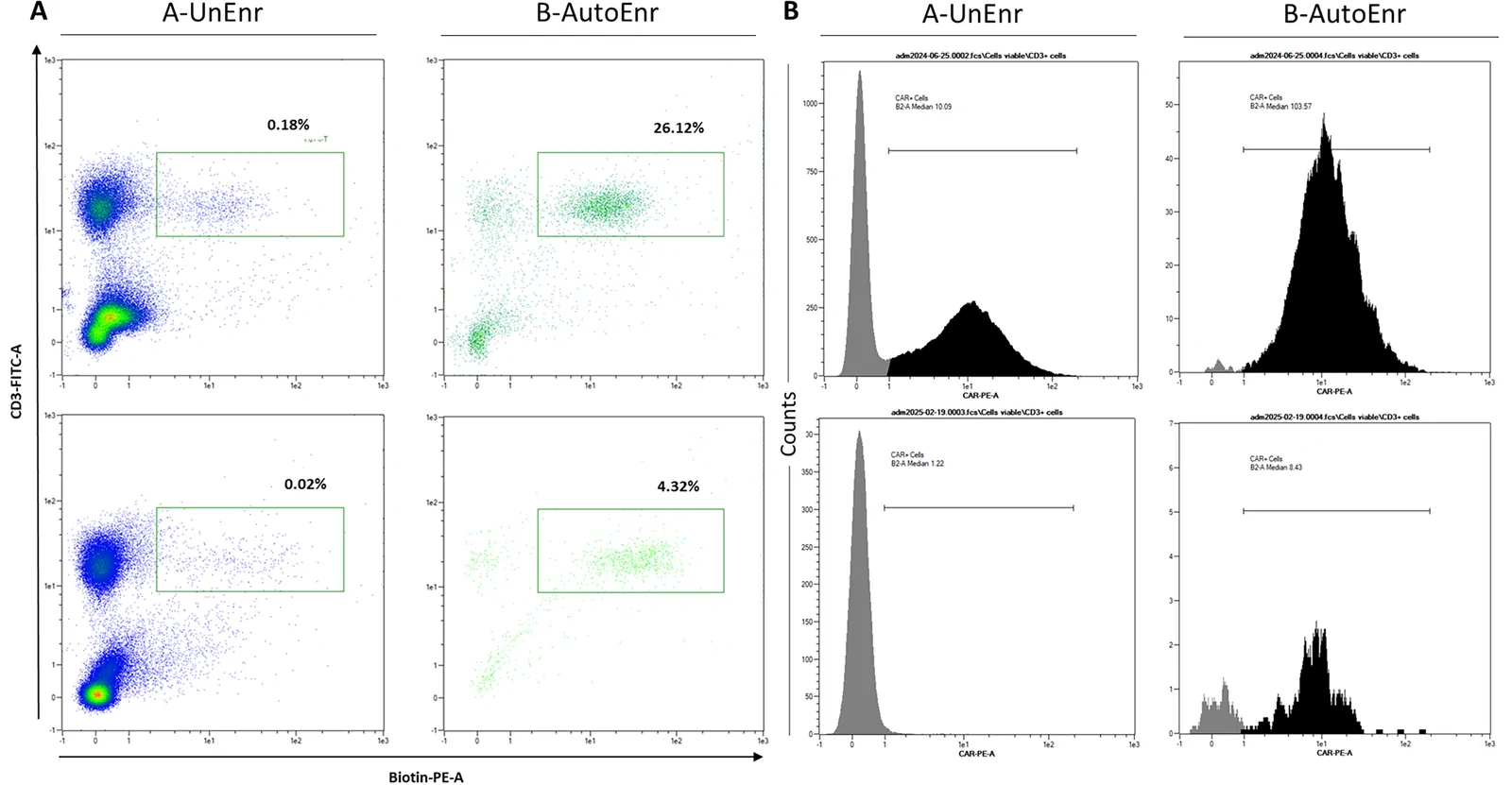
High-Resolution Detection of Low-Frequency CD19-CAR T Cells in Patient Peripheral Blood Following Infusion. Representative flow cytometric dot plots and corresponding fluorescence-intensity histograms illustrate the high-resolution detection of rare circulating CD19-CAR T cells in the peripheral blood of a patient following infusion. By utilizing the EnrichS protocol on the MACSQuant Analyzer in combination with biotinylated detection reagents and PE MicroBeads, target populations that were virtually indistinguishable from background noise under standard acquisition conditions (0.1% and 0.01%; **(A)**, UnEnr) were concentrated into robust, well-defined clusters following automated enrichment (**(A)**, AutoEnr). Frequency values indicate the percentage of CD3/CAR-Biotin-PE^+^ events relative to the total CD3^+^ T-cell population. Fluorescence-intensity histograms of the corresponding gated populations are shown in **(B)**, with fluorescence intensity on the x-axis and event count on the y-axis. Median fluorescence intensity (MFI) values are indicated for the respective populations. Data are representative of two follow-up samples obtained during the analysis of 10 patients.

**Table 4:** Absolute CAR-T cell counts across different frequency ranges.

	Absolute count (Cells/ml)							
Follow-up samples	≥ 10%		1%-10%		0.1-1%		0.01-0.1%	
	CD3+	CAR-T	CD3+	CAR-T	CD3+	CAR-T	CD3+	CAR-T
PT1	938	126.1	3411	94.48	4138	7.45	4038	0.85
PT2	456	62.7	4127	83.78	3976	20.28	3956	0.83
PT3	976	166.1	3615	78.08	3542	22.31	3472	1.11
PT4	1450	154.6	3954	124.16	3746	19.48	3753	1.91
PT5	710	74.3	2832	43.61	4037	7.67	4127	1.28
PT6	1200	136.1	2761	39.21	3067	7.05	3054	0.76
PT7	1635	238.1	2153	68.47	2170	9.11	2265	0.77
PT8	1860	239.4	3254	35.14	4398	32.99	4165	2.37
PT9	2300	230.7	2754	31.95	5320	19.15	4320	0.99
PT10	2105	221.9	5687	81.32	5012	29.07	5459	2.35

Absolute counts of CD3^+^ T cells and corresponding CAR-T cells (cells/mL) were determined in representative follow-up samples using volumetric flow cytometry. Samples were stratified according to the detected CAR-T cell frequency ranges (≥10%, 1–10%, 0.1–1%, and 0.01–0.1%). Absolute CD3^+^ T-cell counts were obtained by direct volumetric analysis of whole blood, and absolute CAR-T cell counts were calculated by multiplying the frequency of viable CAR-T cells within the viable CD3^+^ T-cell population by the corresponding absolute CD3^+^ T-cell count after correction for background staining.

## 4 Discussion

The clinical success of CAR-T cell therapy is heavily dependent on the long-term persistence in patients of the infused cells. However, monitoring these cells as they decline to near-undetectable levels in peripheral blood remains a significant technical challenge. This study demonstrates that the integration of automated immunomagnetic enrichment with multiparametric flow cytometry on the MACSQuant platform provides a superior solution for the sensitive detection of rare CAR-T populations, bridging the gap between its sensitivity of molecular detection by qPCR or ddPCR and its cellular resolution at the level of viability and sub-set identification.

A primary finding of this study is the remarkable increase in sensitivity of detection of CAR-Tcells achieved through the process of its enrichment workflow. In our experiments designed with PBMCs spiked-in with CAR-T cells spanning a four-log range from 10% down to 0.001% CAR-Tcells, we successfully identified both, the anti-CD19 or anti-BCMA CAR-T cells at frequency as low as 0.001%. As the frequency of CAR-T cells decreased, the UnEnr-samples approached a limit of detection at the 0.01% and 0.001% levels in Flow Cytometry assay, where the absolute number of acquired events becomes too low to distinguish true positive cells from background noise. The qPCR data showed the limit of detection of UnEnr CAR-T cells at 0.001% level matching to the data obtained by Flow Cytometry. In contrast, the AutoEnr workflow consistently amplified these trace populations; specifically, at the lowest 0.001% dilution, the target cells were concentrated to 1.66% for CD19- and 1.38% for BCMA- CARs respectively within the analyzed fraction. This represents a nearly 800-fold increase in the signal-to-noise ratio, physically removing over 99.9% of non-target PBMCs to allow for statistically robust population analysis. Without such enrichment, these rare cells are frequently misclassified as “negative,” potentially leading to premature clinical conclusions as loss of CAR-T cell surveillance in a patient. This principle has been successfully applied for detection of functional subsets of low frequency antigen-specific CD4+ and CD8+ T cells (11). The versatility of this platform is further evidenced by its application in characterizing rare circulating follicular T helper cells (cTfh). By targeting the activation marker CD154 (CD40L), researchers successfully isolated rare antigen-specific CD3+CD4+CXCR5+ T cells from Ebola vaccine recipients, enabling a high-resolution characterization of cTfh1, cTfh2, and cTfh17 subsets by using an endogenous staining protocol for the detection of CXCR3 and CCR6 (12, 13). Therefore, the automated immunomagnetic enrichment workflow represents a robust and unbiased tool for converting results from the rare, “invisible” immune populations into actionable clinical data. Furthermore, the remarkable consistency between the CD19- and BCMA- CAR expressing constructs suggests that this platform is highly versatile and independent of the specific CAR-antigen affinity. By shifting a near-invisible 0.001% population into a clearly quantifiable >1% range, this method improves the limit of detection compared with the sensitivity of genomic detection of CAR-Tcells by molecular assays such as qPCR or digital droplet PCR (ddPCR) (14, 15), and next generation sequencing (NGS) (16) which played an important role in tracking CAR-T cell kinetics and minimal residual disease following CAR-T therapy. Importantly, the integration of automated magnetic enrichment directly with MACSQuant flow cytometry offers practical advantages for clinical and translational laboratories. Automation reduces operator-dependent variability, improves reproducibility, and facilitates standardized workflows compatible with routine monitoring of post-infusion circulating CAR-T cells in blood samples of patients in making clinical decisions and helping patients’ care. The ability to directly acquire enriched samples on the same platform supports rapid turnaround times while preserving multiparametric analytical capability. This is particularly relevant during the contraction and persistence phases of CAR-T cell kinetics, when circulating CAR-T cells may be present at very low levels yet remain functionally relevant for long-term tumor surveillance.

qPCR was also employed as an additional analytical technique in this study. Molecular methods such as qPCR are highly sensitive and effective for detecting rare CAR-T cell populations through vector copy number (VCN) analysis. However, these assays detect transgene DNA rather than intact, viable cells and provide no information on cellular phenotype or functional status (17). During later stages of CAR-T cell persistence, qPCR signals may originate from residual transgene DNA derived from apoptotic or transcriptionally inactive cells. In contrast, flow cytometry detects surface CAR expression on viable cells, providing direct assessment of the therapeutically active cell population. Furthermore, qPCR requires transgene-specific primers and involves multiple processing steps, resulting in longer turnaround times. By comparison, flow cytometry enables same-day analysis with single-cell resolution, supporting timely monitoring of patients following CAR-T cell infusion.

As a novel aspect of this study and with patients’ informed consent, we validated the clinical proof-of-concept of this automated immunomagnetic enrichment workflow through the analysis of samples obtained from patients enrolled in the ADSCC CAR-T cell therapy program following CD19-CAR-T cell infusion (18, 19). Consistent with the results observed in the controlled spike-in experiments, the platform produced a marked and reproducible enhancement of the detectable CAR-T cell signal across a broad range of baseline abundances, demonstrating that the enrichment workflow maintains high sensitivity and precision within the complex biological environment of post-infusion patient samples. These findings support the feasibility of implementing this methodology in a clinical setting for the detection and monitoring of rare CAR-T cell populations and provide a foundation for future studies evaluating its broader clinical utility in longitudinal patient monitoring.

Despite the promising findings presented herein, several limitations should be acknowledged. The current study was designed primarily as a technical proof-of-concept to evaluate the feasibility and analytical performance of combining automated immunomagnetic enrichment with flow cytometric analysis on a single platform for the sensitive detection of rare CAR-T cell populations. Consequently, the patient cohort was relatively small and was not intended to support broad clinical conclusions regarding CAR-T cell persistence kinetics or treatment outcomes. Nevertheless, the workflow demonstrated a significant improvement in the detection of low-frequency CAR-T cells while minimizing hands-on manipulation and operator-dependent variability, highlighting its potential applicability in routine clinical laboratory settings. While the current platform enables highly sensitive detection and characterization of viable rare CAR-T cell populations, future studies involving larger and more diverse longitudinal patient cohorts are required to establish correlations between CAR-T cell persistence, phenotypic profiles, and clinical outcomes. Furthermore, expanding the assay beyond descriptive phenotyping to include functional characterization through the assessment of exhaustion markers, differentiation markers, activation markers, lineage markers, cytokine production, and anti-tumor cytotoxic activity may provide additional insights into the mechanisms governing CAR-T cell persistence, functionality, and exhaustion. In addition, the enrichment-coupled flow cytometry workflow enables same-day detection of viable CAR-T cells with minimal sample manipulation while providing single-cell resolution and detailed phenotypic characterization. Beyond transgene detection with molecular approaches such as qPCR or ddPCR, the platform allows simultaneous assessment of CAR-T cell frequency, viability, and phenotype, thereby providing a more comprehensive evaluation of CAR-T cell persistence and immune reconstitution in patients. Ongoing studies involving substantially larger patient cohorts from both manufacturing and clinical settings are currently underway to further evaluate the clinical utility of this approach through longitudinal monitoring and correlation with relevant clinical parameters.

In conclusion, this study describes a novel methodology for the detection of rare CAR-T cells following adoptive cellular immunotherapy. The results demonstrate that automated immunomagnetic enrichment selectively concentrates CAR-T cells and substantially enhances the detectable signal of rare populations without increasing non-specific background events. This improved sensitivity enables the reliable identification of low-frequency viable CAR-T cells and may provide an early indication of CAR-T cell persistence in peripheral blood. Furthermore, the workflow preserves single-cell resolution and phenotypic characterization while requiring minimal hands-on manipulation and providing rapid turnaround times. Although additional clinical validation in larger patient cohorts is required, the methodology can be further optimized and expanded to support routine clinical monitoring of CAR-T cell persistence, immune reconstitution, and treatment response in patients receiving CAR-T cell therapy.

## Data Availability

The original contributions presented in the study are included in the article/**Supplementary Material**. Further inquiries can be directed to the corresponding author.
