# Supplementary material for: Magnetic enrichment–coupled flow cytometry for highly sensitive detection of persistent CAR-T cells in spiked and clinical peripheral blood samples
Source: Front Immunol. 2026 Oct 21;17:1872290. doi: 10.3389/fimmu.2026.1872290 (PMC13731331; doi:10.3389/fimmu.2026.1872290)
Supplement: Supplementary file 1 [file Table1.docx]

| **Workflow Step** | **Standard Acquisition (UnEnr)** | **EnrichS Workflow** |
| --- | --- | --- |
| **Sample uptake volume** | 100 µL | 400 µL |
| **Priming column** | – | 2 min 45 s |
| **Enrichment, loading and washing** | – | 11 min 03 s |
| **Cleaning tubes** | – | 57 s |
| **Elution** | – | 1 min 13 s |
| **Sample acquisition** | 2 min 00 s | 8 min 02 s |
| **System washing** | 25 s | 57 s |
| **Column cleaning** | – | 3 min 00 s |
| **Final column priming** | – | 2 min 45 s |
| **Sample loading/preparation** | 12 s | Included in enrichment workflow |
| **Total processing time** | 2 min 37 s | 30 min 13 s |

**Supplementary Table S1. Workflow steps, sample uptake volumes, and processing times for standard acquisition (UnEnr) and automated immunomagnetic enrichment (EnrichS) on the MACSQuant Analyzer 10.** **Abbreviations:** UnEnr, standard flow cytometric acquisition without enrichment; EnrichS, automated magnetic enrichment workflow integrated into the MACSQuant Analyzer 10.
