# Supplementary material for: Magnetic enrichment–coupled flow cytometry for highly sensitive detection of persistent CAR-T cells in spiked and clinical peripheral blood samples
Source: Front Immunol. 2026 Oct 21;17:1872290. doi: 10.3389/fimmu.2026.1872290 (PMC13731331; doi:10.3389/fimmu.2026.1872290)
Supplement: Supplementary file 2 [file Table2.docx]

| **Parameter** | **CD19 CAR-T** | **BCMA CAR-T** |
| --- | --- | --- |
| Mean blank signal (%) | 0.000075 | 0.000069 |
| Limit of Blank (LOB, %) | 0.000241 | 0.000241 |
| Functional LOD (%) | 0.001 | 0.001 |
| Functional LOQ (%) | 0.001 | 0.001 |
| LOD (cells in 10 × 10⁶ PBMCs) | 100 | 100 |
| LOD (ratio) | 1:100,000 | 1:100,000 |
| Precision at LOQ (CV, %) | 3 | 2.2 |
| Mean viability after enrichment (%) | 95.34–98.76 | 95.34–98.76 |
| Background signal in blank controls | Negligible | Negligible |

**Supplementary Table S2. Analytical sensitivity, specificity, and precision of the automated immunomagnetic enrichment workflow for CD19- and BCMA-directed CAR-T cell detection.** The limit of blank (LOB) was calculated from CAR-negative PBMC control samples. Functional limit of detection (LOD) and limit of quantification (LOQ) were established at the lowest tested concentration (0.001%), corresponding to approximately 100 CAR-T cells in 10 × 10⁶ PBMCs (1 CAR-T cell per 100,000 PBMCs). Precision at the LOQ was determined using the coefficient of variation (CV).
